#  ‎ Factors Affecting the Choice of Psychiatry as a Specialty in ‎Psychiatry Residents in Iran

**Published:** 2016-07

**Authors:** Seyed Saeed Sadr, Razieh Nayerifard‎‎, Seyed Mehdi Samimi Ardestani, Massood Namjoo

**Affiliations:** 1 Behavioral Sciences Research Center, Shahid Beheshti University of Medical Sciences, Tehran, Iran.; 2 Department of Psychiatry, Imam Hossein Hospital, Shahid Beheshti University of ‎Medical Sciences, Tehran, Iran.

**Keywords:** *Choice*, *Psychiatry*, *Resident*, *Specialty*

## Abstract

**Objective: **The aim of this study was to investigate the current factors affecting the choice of ‎psychiatry as a specialty and to detect the main factors in their choice.‎

**Method: **This descriptive study included 75 first year psychiatry residents in the academic year of ‎‎2014/2015. A Likert-type anonymous questionnaire consisting of academic and ‎demographic data with 43 questions, which evaluated the reason for choosing ‎psychiatry as a specialty, was given to the residents.‎

**Results: **The participants had a positive opinion about 28 items of the questionnaire, meaning that ‎these items had a positive effect in choosing psychiatry as a specialty (questions with P ‎value less than 0.05 and a positive mean). More than 80% of the residents had a positive ‎opinion about six items of the questionnaire (amount of intellectual challenge, variety of ‎knowledge fields relevant to psychiatry, emphasis on the patient as a whole person, the ‎importance of treating mental illnesses in the future, work pressure and stress of the ‎field during residency and coordinating with the person's life style). The participants ‎had a negative opinion about two items of the questionnaire (questions with a P value ‎less than 0.05 and a negative mean). They included experiencing mental illness ‎personally through relatives or close friends as well as the income in psychiatry. ‎Moreover, 36% of the residents with a more definite opinion mentioned that they chose ‎psychiatry as a specialty because of the limitations in residency exam.‎

**Conclusion: **Assistants had a positive opinion about most of the questions and this positive attitude ‎seemed to be an important factor in their specialty choice. However, attending to the ‎preventing factors may increase the selection of psychiatry as a specialty.‎

Psychiatry is facing a deficiency of specialists, named “recruitment crisis” (1). Many ‎researchers in different countries witnessed negative attitudes of medical students ‎toward psychiatry and the small number of medical students choose psychiatry as their ‎future career (2). A number of medical students have no interaction with patients with ‎mental illness before their presence in the psychiatry ward, and some of them believe ‎that working in this field is stressful (3, 4).‎

Recently, many studies have been conducted on the factors affecting the selection of ‎psychiatry as a specialty (5). Evidence shows that students consider psychiatry as an ‎interesting medical discipline, whereas they believe this career has a low socioeconomic ‎level among other specialties (6). Attitude toward psychiatry has an impact on ‎individuals working in this field and treatment of mental patients (7). ‎

There are a few researches on the perception of psychiatry residents and specialists (8-9).‎

In Iran, medical training lasts at least six years and medical students usually undertake a ‎one-month psychiatry clerkship in the fourth or fifth year of training as well as ‎approximately 20 hours of theoretical course. In 2014, the number of psychiatrists was ‎‎1500-1700 in Iran, indicating a considerable deficiency in this area (10).‎

Previous studies reported a decline in interest of medical students about working in ‎psychiatry (11). Some studies have shown a positive attitude toward psychiatry as a ‎subject and choosing it as a career among preclinical medical students (12, 13). Some ‎researchers believe that a better attitude would not necessarily lead to the choice of ‎psychiatry as a specialty (2).‎

Medical education theorists have several justifications about psychiatric situation (14). ‎They have linked the reluctance of medical students to psychiatry with various factors ‎including biological emphasis of psychiatry in recent years, no economic justification for ‎this field compared to other medical specialties and even unfair competition of social ‎workers and psychologists with psychiatrists, inhibitor thoughts of colleagues and non-‎psychiatrist consultants (15, 16). Approximately 3% of all medical students choose ‎psychiatry as a career (17) and individuals with positive attitude to mental health who ‎select psychiatry are about three times more (18). In this study, we aimed to investigate ‎factors affecting the choice of psychiatry in psychiatry residents instead of medical ‎students.‎ ‎

## Materials and Method

An anonymous 43- item Likert-type questionnaire was prepared using the questionnaire ‎of the Galeazzi et al. study (5). We added the following five items to the questionnaire: ‎Work pressure and stress of field during residency; work pressure and stress of field ‎after graduation; the legal complaint of psychiatry; coordination with the person's life ‎style; and limitations in choosing specialty because of residency exam. The questionnaire ‎also contained academic and demographic data and was translated into Farsi with ‎forward/backward method.‎

‎ Content validity was confirmed by five faculty members of the Department of ‎Psychiatry at Imam Hossein Hospital, Tehran, Iran and by a pilot study on 10 cases of ‎psychiatry residents. Using Cronbach alpha, reliability calculated to be 0.872, ‎representing a high internal consistency. ‎

In the questionnaire, participants were asked to rate each of the 43 items attributed to ‎psychiatry as a specialty on a 5-point Likert scale. The scale ranges from –2, indicating ‎that the factor had a very negative effect on their choice, meaning that they felt that the ‎aspect had led them away from choosing psychiatry as a specialty to +2, indicating that ‎the quality of item attributed to the discipline exerted a very positive effect on their ‎decision to choose postgraduate training in psychiatry. Completion of the questionnaire ‎took approximately 20 minutes.‎

The sample size to estimate the average score given to the questionnaire items, with ‎pooled standard deviation (SD) 0.67, precision 0.15, and Type I error 0.05 and power of ‎the test 80% determined 75 persons. Considering 20% attrition, questionnaires were ‎distributed to 90 residents studying in the first year of psychiatry. The questionnaire was ‎anonymous, and its completion was voluntary; returning a completed questionnaire was ‎considered as an informed consent.‎

Out of the 90 participants, 77 returned the questionnaires and two were excluded from ‎the analysis due to missing data. Multi-stage sampling was conducted‏.‏‎ The ‎questionnaires were distributed to the participants by one of the authors at Shahid ‎Beheshti University. In other centers, after coordination with a faculty member of the ‎department of psychiatry, a professor delivered and collected the questionnaires after ‎the residents completed them.‎

Mean, SD, frequency and percentage were used to describe the data. One- sample t- test ‎was used to examine the role of the questions in choosing psychiatry as a specialty. The ‎mean of each question was compared to zero, and if the average of the relevant question ‎was significantly different from zero, it meant that the question was involved in this ‎selection. Mann-Whitney test was used to examine the role of gender on scores from ‎each of the questions. Data were analyzed using the Statistical Package for the Social ‎Sciences (SPSS) software version 20. The Ethics Committee of Shahid Beheshti ‎University of Medical Sciences (SBUMS) approved this study.‎

## Results

There were 30 (40%) respondents at Tehran universities including Tehran University of ‎Medical Sciences (TUMS), Shahid Beheshti University of Medical Sciences (SBUMS), ‎Iran University of Medical Sciences (IUMS), and University of Social Welfare and ‎Rehabilitation Sciences (USWRS) as well as 45 (60%) respondents at Mashhad, Tabriz, ‎Shiraz, and Isfahan universities.‎

The participants included 17.6% males and 82.4% females, of whom, 82.4 and 17.6% ‎were married and single, respectively. The average (±SD) age of the residents was 30.3 ‎‎(±4.09) years (Table1). The mean (±SD) final score at the medical school was ‎‎16.38 (±0.94) and the mean (±SD) score of residency exam was 321.11 (±30.55) (Table1). The minimum times of taking the residency exam was one time and the ‎maximum was five times; and the frequency was 46.4 and 4.3%, respectively.‎

‎ Participants had a positive opinion about the 28 items of the questionnaire, which ‎means that the above factors had a positive effect on choosing psychiatry as a specialty; ‎i.e., questions with p value <0.05 and positive mean (Table 2). Six prominent features ‎which persuaded residents to select psychiatry were as follows: Amount of intellectual ‎challenge (N = 58); variety of knowledge fields relevant to psychiatry (N = 62); ‎emphasis on the patient as a whole person (N = 62); importance of treating mental ‎illnesses in the future (N = 65); work pressure and stress of the field during residency (N ‎‎ = 60); and coordination with the life style of the person (N = 63) (items 1, 3, 4, 36, 39, ‎and 42).‎

The participants had a negative opinion about two items of the questionnaire, which ‎indicates that these factors had a negative effect on their choice; i.e., questions with p ‎value <0.05 and negative mean. These include experience of mental illness personally or ‎by relatives or close friends (N = 35) and salary (N = 43) (items 7, 23). Moreover, 36% ‎of the residents chose psychiatry due to limitations in their residency exam (item 43).‎

In terms of examining gender role in the response to each of the questionnaire items, ‎female participants had a positive opinion about three items of the questionnaire ‎‎[Questions with P value <0.05 and female median (IQR)> male median (IQR)]. 

**Table1:** Baseline Characteristics of Psychiatry Residents

**Characteristic**		**Mean**	**SD, Range**
**Age (years)**		30.3	4.09, 25-46
		N*	%
**Sex**			
	Female	61	84.4
	Male	13	17.6
**Marital status**			
	Single	13	17.6
	Married	61	84.4
**Final score at medical school (Maximum 20)**		16.38	0.94, 14.14- 18.70
**Score at residency exam(Maximum 600)**		321.11	30.55, 119-380

* Total does not equal 75 because data were missing for a respondent

**Table2:** Current Factors Affecting the Choice of Psychiatry as a Specialty in Psychiatry residents

	**Question**	**Mean** *	**95% CI**	**P value** **
1	Amount of intellectual challenge	1.083	(0.87,1.29)	<0.001
2	Opportunity for cultivating interest in humanities	0.905	(0.62,1.2)	<0.001
3	Variety of knowledge fields relevant to psychiatry	1.213	(1.01,1.42)	<0.001
4	Emphasis on the patient as a whole person	1.333	(1.12,1.55)	<0.001
5	Original and unique themes encountered in psychiatry	1.12	(0.91,1.33)	<0.001
6	Curiosity about and attraction to the topic ‘‘madness’	0.365	(0.1,0.63)	0.008
7	Experience of mental illness personally or by relatives or close friends	-0.52	(-0.8,-0.24)	<0.001
8	Experience of psychological problems personally or by relatives or close friends	-0.162	(-0.46,0.14)	0.284
9	Opportunity to know unexplored aspects of self by working with patients	0.88	(0.63,1.13)	<0.001
10	Contact or close acquaintance with psychiatrists	0.28	(0,0.56)	0.052
11	Importance of social and relational issues in psychiatry	1.093	(0.9,1.29)	<0.001
12	Intensity or quality of emotional contact with psychiatric patients	0.176	(-0.01,0.36)	0.068
13	Importance of narratives and meanings more than significance of technology	0.351	(0.1,0.6)	0.006
14	Opportunity to practice psychotherapy	1	(0.76,1.24)	<0.001
15	Opportunity for long-term relationships with patients	0.093	(-0.14,0.33)	0.429
16	Contact with psychiatric patients during medical school	0.12	(-0.13,0.37)	0.343
17	Global quality of psychiatric teaching at medical school	0.56	(0.33,0.79)	<0.001
18	Teaching style and personality of professors of psychiatry	0.96	(0.72,1.2)	<0.001
19	Themes of psychiatric lectures	0.827	(0.63,1.02)	<0.001
20	Contact with psychiatric residents	0.427	(0.18,0.68)	0.001
21	Opportunity for complete use of medical training	0.253	(0.04,0.47)	0.021
22	Global opportunity for employment after specialty	-0.013	(-0.31,0.28)	0.928
23	Salary in psychiatry	-0.653	(-0.92,-0.38)	<0.001
24	Range of different employment opportunities after specialty	0.067	(-0.2,0.33)	0.619
25	Status of psychiatry among peers	-0.08	(-0.35,0.19)	0.552
26	Status of psychiatry within the medical faculty	-0.053	(-0.32,0.21)	0.686
27	Status of psychiatrists in society	-0.053	(-0.32,0.22)	0.695
28	Status of psychiatry among family and friends	-0.227	(-0.52,0.07)	0.129
29	Stigma of mental illness	-0.267	(-0.55,0.01)	0.061
30	Prestige of the department of psychiatry or the university chosen for training	0.533	(0.3,0.76)	<0.001
31	Pace of development in psychiatry as a field	0.893	(0.66,1.13)	<0.001
32	Progress in the biological study and treatment of mental illnesses	0.905	(0.68,1.13)	<0.001
33	Recent developments of psychiatry as a neuroscience	1	(0.79,1.21)	<0.001
34	Opportunities for research	0.8	(0.54,1.06)	<0.001
35	Level of evidence base of psychiatry	0.514	(0.3,0.72)	<0.001
36	Importance of treating mental illnesses in the future	1.307	(1.1,1.51)	<0.001
37	Efficacy of psychiatric treatments	0.76	(0.54,0.98)	<0.001
38	Range of psychiatric therapies	0.712	(0.48,0.95)	<0.001
39	the work pressure and stress of field during residency	1.187	(0.97,1.4)	<0.001
40	the work pressure and stress of field after graduation	1.067	(0.85,1.29)	<0.001
41	The legal complaint of psychiatry	0.419	(0.15,0.69)	0.003
42	Coordination with the person's life style	1.333	(1.14,1.53)	<0.001
43	Limitations in choosing specialty because of residency exam’s score	-0.093	(-0.4,0.22)	0.55

* Responses were scored on a 5-point Likert scale ranging from –2 (very negative factor) to +2 (very positive factor).

** P value based on one sample t-test

They ‎included importance of social and relational issues in psychiatry, work pressure and ‎stress of the field during residency, coordination with the person's life style (items 11, ‎‎39 and 42).‎

The median in the female and male groups was 1 vs. 0.5 in question 11, 2 vs. 0 in ‎question 39 and 2 vs. 0 in question 42, respectively.‎

## Discussion

This study was the first type in its kind in Iran, which tried to determine the current ‎factors affecting the choice of psychiatry as a specialty in psychiatric residents to detect ‎the factors affecting recruitment into psychiatry as a medical specialty.‎

According to the results in this study, 82.4% of the participants were female; this rate ‎was 75% in an Italian study (5), 80% in Romania (19) and 68.3% in Turkey (34). In our ‎study, 36% of the residents with more definite view mentioned that they chose ‎psychiatry as a specialty due to limitations in the score of residency exam. However, ‎Voinescu et al. reported that the majority of junior doctors in psychiatry (71%, n = 44) ‎believed that many trainees who had not been able to obtain a position in other ‎specialties eventually entered psychiatry (19).‎

In our study, participants had a positive opinion about 28 items of questionnaire, which ‎was more than other studies (5, 34).Some studies named psychiatry as a stressful career ‎leading the students not to choose psychiatry as a specialty (20). However, in our study, ‎the majority of the residents had a positive opinion about the work pressure and stress of ‎the field during residency and after graduation.‎

In previous studies, psychiatrists’ discontent due to low income and negative attitude of ‎the society to psychiatry adopted the attitude of medical students to psychiatry and ‎made their decision about specialty selection (15, 21-25). Some of the researchers ‎believe that the economic situation of the students play an important role in their ‎decision to choose a specialty (26), and choosing a field may be affected by some ‎factors such as income (27, 34). In this study, salary in psychiatry had a negative effect ‎in participants’ choice. In Ozer et al. study, teaching style and personality of professors ‎of psychiatry were the most positively rated items affecting the choice of psychiatry ‎‎(34) in contrast to the findings of Italian study (5); therefore, this point should be ‎considered by educators who do not believe in the impact of their actions on student’s ‎perceptions (Factor 18).‎

Our analysis showed that the main motivation for choosing psychiatry as a specialty in ‎these residents seemed to be the curiosity distributed across the different levels of the ‎field: Interest in the utility of the relational and affective interaction with patients ‎‎(Factors 4 and 5); fascination with the mystery of mental illness (factor 6); obvious ‎desire for knowledge (Factor 1) and various aspects of psychiatry such as ‎psychotherapy; and the narrative perspective of the discipline and a variety of specific ‎interventions (Factors 3, 13, and 38). Galeazzi et al. (5) showed that future psychiatrists ‎might have a greater desire for a deep emotional contact with patients (Factor 12); ‎however, this was not significant in our study.‎

Amini et al. (28) found that psychiatric internship cannot persuade more students to ‎choose psychiatry as a possible career and it seems that psychiatric training in Iran does ‎not positively affect students’ attitude to psychiatry. However, in our study, the global ‎quality of psychiatric education at medical school (Item 17) as well as teaching style and ‎personality of professors of psychiatry (Item 18) had a positive influence on ‎participant’s choice.‎

## Limitations

This study had some limitations. One limitation was related to the nature of the ‎questions. Although the questionnaire was translated consistent with scientific ‎principles, the level of understanding the questions in the participants might have been ‎different as an inevitable human error. ‎

The second limitation was that the participants` response might have been affected by a ‎few months of residency. Another limitation was that psychiatry residents are only a ‎small part of the individuals who have chosen the field of psychiatry. Some of them ‎were not admitted at all and some had been accepted in other fields. ‎

Therefore, our results cannot be generalized to the entire medical students who have ‎selected this career. We did not monitor changes in attitudes during residency; however, ‎some studies report the modification of medical students’ attitudes during medical ‎education (29-33). Furthermore, the participants were psychiatry residents, not first-year ‎or midcourse medical students, who are less certain about their future training choices.‎

## Conclusion

The findings of this study revealed that psychiatry residents had a positive opinion ‎about most of the questions and this positive attitude seemed to be the important factor ‎in their choice of specialty. However, attending to the preventing factors may increase ‎the selection of psychiatry as a specialty.‎
